# Unique Case Reports Associated with Ovarian Failure: Necessity of Two Intact X Chromosomes

**DOI:** 10.1155/2012/640563

**Published:** 2012-04-11

**Authors:** Lakshmi Rao Kandukuri, Venkata Padmalatha, Murthy Kanakavalli, Raseswari Turlapati, Mangalipally Swapna, Metuku Vidyadhari, Govindaraghavan Saranaya, Kattera Himaja, Mamata Deenadayal, Bipin Kumar Sethi, Prasun Deb, Nalini Gupta, Baidyanath Chakraborthy, Pratibha Nallari, Lalji Singh

**Affiliations:** ^1^Clinical Research Facility-Medical Biotechnology, Centre for Cellular and Molecular Biology, Annexe II, Hyderabad 500007, India; ^2^Genome Foundation, Centre for Cellular and Molecular Biology, Hyderabad 500007, India; ^3^Infertility Institute and Research Centre, Secunderabad 500063, India; ^4^Tapadia Diagnostic Centre, Hyderabad 500029, India; ^5^Krishna Institute of Medical Sciences, Hyderabad 500016, India; ^6^Institute of Reproductive Medicine, Kolkata 700064, India; ^7^Department of Genetics, Osmania University, Hyderabad 500007, India

## Abstract

Premature ovarian failure is defined as the loss of functional follicles below the age of 40 years and the incidence of this abnormality is 0.1% among the 30–40 years age group. Unexplained POF is clinically recognized as amenorrhoea (>6 months) with low level of oestrogen and raised level of Luteinizing Hormone (LH) and Follicle Stimulating Hormone (FSH > 20 IU/l) occurring before the age of 40. It has been studied earlier that chromosomal defects can impair ovarian development and its function. Since there is paucity of data on chromosomal defects in Indian women, an attempt is made to carry out cytogenetic evaluation in patients with ovarian failure. Cytogenetic analysis of women with ovarian defects revealed the chromosome abnormalities to be associated with 14% of the cases analyzed. Interestingly, majority of the abnormalities involved the X-chromosome and we report two unique abnormalities, (46,XXdel(Xq21-22) and q28) and (mos,45XO/46,X+ringX) involving X chromosome in association with ovarian failure. This study revealed novel X chromosome abnormalities associated with ovarian defects and these observations would be helpful in genetic counseling and apart from, infertility clinics using the information to decide suitable strategies to help such patients.

## 1. Introduction

Ovarian failure reflects spectrum of symptoms, which include amenorrhea, gonadal dysgenesis, and premature ovarian failure (POF). Amenorrhea can be primary or secondary. POF is defined as the loss of functional follicles below the age of 40 years. The incidence of this abnormality is 0.1% among the 30–40 years age group. It keeps increasing and reaches up to 1% among the 40 years age group [1]. The diagnosis of unexplained POF is clinically recognized as amenorrhoea (>6 months) occurring before the age of 40 with low level of oestrogen and raised level of luteinizing hormone (LH) and follicle-stimulating hormone (FSH > 20 IU/l) [2]. Different rationales based on immunological abnormalities [3], viral infections [4], cytotoxic drugs [5], physical stress, nutrient deficiency, chemotherapy, and radiation effects [6] have been advanced to explain POF. The crucial factor also includes the important role of chromosomes in maintaining the normal ovarian development and function. It has been studied earlier that chromosomal defects can impair ovarian development and its function [7]. The most frequent phenotype associated with sex chromosomal defects is Turner's syndrome in which there is only one X chromosome instead of two. Most of the women with primary amenorrhea possess Turner's or its variant or have a Y chromosome in the cells [8]. In the women with secondary amenorrhea the chromosome defects like loss of entire X chromosome or, any gross chromosomal rearrangements are not generally noticed [9]. There is considerable variation among the chromosomal abnormalities that cause ovarian failure in different populations. Since there is paucity of data on chromosomal defects in Indian women, we have made an attempt to carry out cytogenetic evaluation of patients with ovarian failure.

## 2. Materials and Methods

### 2.1. Subjects

A total of 457 women, 230 with POF, 157 with primary amenorrhea, and 73 with secondary amenorrhea were subjected to this study. The Institutional Review Board approved the study. Subjects were referred from infertility clinics/hospitals of Hyderabad, India, after a thorough clinical evaluation by gynecologists. Complete clinical assessment and information pertaining to medical and gynecological history, age at menses, menstrual history, age at menopause and hormonal levels of every case were recorded in specially designed proformas. Following the definitions, selection of subjects was done for primary, secondary amenorrhea and POF (associated with high gonadotrophin level, that is, FSH > 40 IU/l detected from two different serum samples at 1 month interval). Of the 457 women, among the various defects detected, 2 showed unique clinical features and karyotypes. The detailed case history of these two women is given below.

**Case 1**

**Figure 1:**
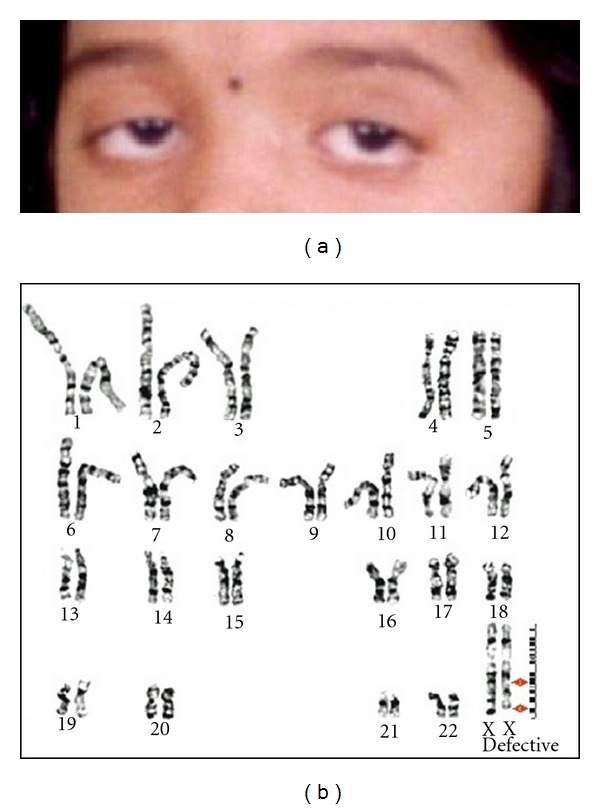
(a) Photograph of the patient with BPES showing peculiar ocular deformities. (b) GTG-banding results showing two break points on one of the X chromosome.

A 17-year-old girl was referred with a history of secondary amenorrhea. The girl had attained menarche at the age of 11 years and complained irregular menstrual cycles since one year. Her medical and family history had no significant abnormalities. She was healthy with average height and weight with normal limbs, and had a body mass index of less than 20. Her intellectual capabilities were also found to be normal. Clinical investigation showed normal secondary sexual characteristics. The girl had no cardiac, respiratory and neurological abnormalities. Abdominal and per vaginal examinations revealed normal findings. Endocrinological studies showed normal serum FSH, LH, prolactin and thyroid stimulating hormone (TSH) levels. However, peculiar deformity of the eyelids was seen consisting of telecanthus, blepharophimosis, ptosis and epicanthus inversus (BPES) with a palpebral fissure length less than 2.5 cm (Figure 1(a)).

**Case 2**

The proband was born after a normal labour and is the daughter of healthy and nonconsanguineous parents. She has two brothers and two sisters who are normal. Her phenotype, intellectual development were normal and allowed a professional career. At the age of 15 she was first presented as an outpatient in endocrine clinic because of primary amenorrhea and short stature. Physical examination showed her height to be 140 cm while her weight was only 30 kg. Puberty stage according to Tanner was 3. Clinical examination showed disproportion of the body with short neck, short limbs, shield chest, low posterior hair line, and cubitus valgus. Skeletal maturity was abnormal for her age, which corresponded to the age of 11 years. The endocrinological examination revealed elevated levels of serum FSH 65 mIU/mL (normal 3–20 mIU/mL), LH of 20 mIU/mL (0.8–10.4 mIU/mL), and a reduced E2 level of 4.5 pg/mL (10–77 pg/mL). External genitalia were normal and a pelvic ultrasound showed no visualization of ovaries and fallopian tubes but showed a small uterus.

### 2.2. Cytogenetic Analysis

About 2 mL of peripheral blood was collected from the 457 women with their informed written consent. All the samples were subjected to lymphocyte culture [10]. Fifty GTG-banded [11] metaphase chromosomes were examined for each case. In cases with abnormalities, a total of 100 metaphases were analyzed.

### 2.3. Fluorescence In Situ Hybridization (FISH) and Microscopy

FISH was performed, wherever it was necessary, on metaphase spreads derived from patient's lymphocytes. Hybridizations were performed using DNA probes specific for X chromosome from (Vysis, Naperville, IL) according to the Vysis manufacturer's protocol. The probes used in this study were Whole Chromosome Paint for chromosome X (WCP X) SpectrumGreen, *L*ocus-*S*pecific *I*dentifier (LSI) SpectrumOrange Androgen Receptor DNA probe, Mixtures 1 and 2 from ToTelVysionTM multicolor-color DNA Probe Mixtures. *Mixture 1* is TelVysion 1p SpectrumGreen, TelVysion 1q SpectrumOrange, TelVysion Xp/Yp SpectrumOrange and SpectrumGreen, and CEP X Spectrum Aqua. *Mixture 2* is TelVysion 2p SpectrumGreen, TelVysion 2q SpectrumOrange, TelVysion Xq/Yq SpectrumOrange and SpectrumGreen, CEP X SpectrumAqua. After following the procedural steps with independent probe hybridizations, slides were counterstained with 20 *μ*L of 4–6 diamino-2-phenylinodole (DAPI) (10 g/mL). Bright light and fluorescence microscopy was performed with a Zeiss Axioscope microscope (Zeiss, Jena, Germany). A dual filter set (Vysis, Naperville, IL) was used for simultaneous detection of DAPI, SpectrumOrange, and SpectrumGreen signals. Several images were processed using Cytovision-automated system (Applied Imaging, Santa Barbara, CA). Each slide was scored by at least three observers.

## 3. Results

Cytogenetic analysis of 457 women with primary, secondary amenorrhea, and POF revealed 14% having chromosome defects. Among them, 53 individuals showed X-chromosome abnormalities as summarized in Table 1. Very few autosomal defects were noted wherein chromosome 2, 3, 4, 9, 15, and 19 are involved. Of the X chromosome abnormal karyotypes, two were unique. One had two structural abnormalities, a large interstitial deletion spanning Xq21.1-Xq22.2 and terminal deletion in Xq28 on the same X chromosome (Figure 1(b)).

**Figure 2:**
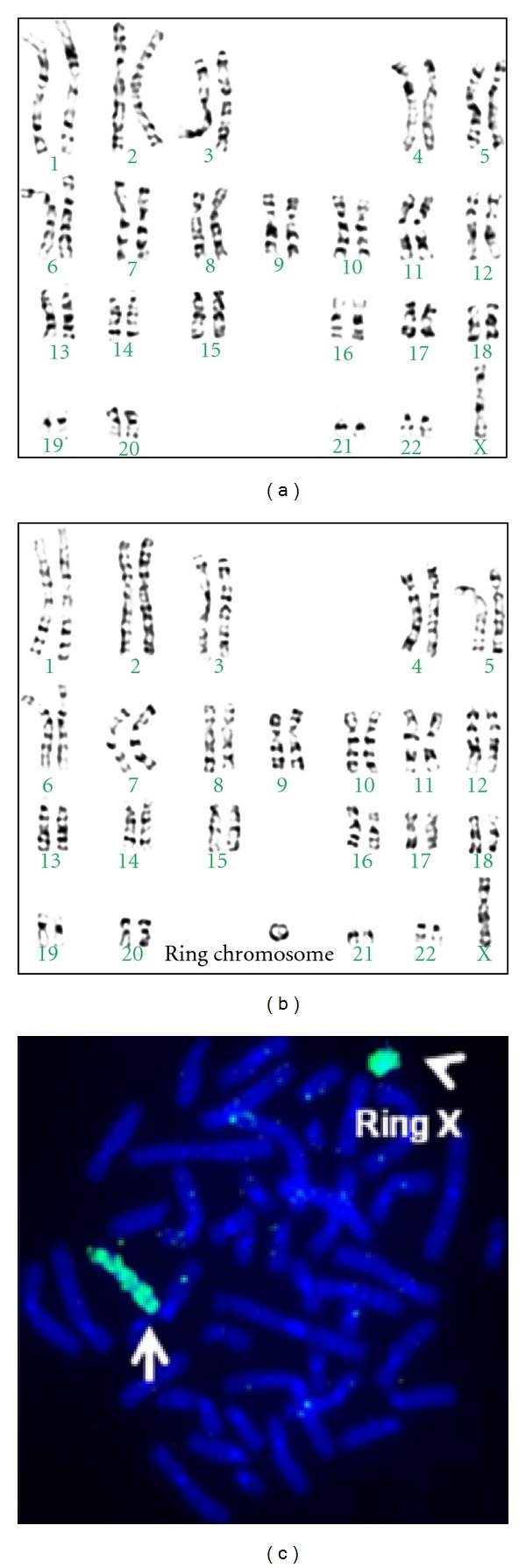
GTG-banding results showing mosaicism with two different cell lines of a Turner's variant (a) 45,XO karyotype and (b) 46,X+ringX karyotype. (c) FISH of metaphase spread with the Vysis WCP DNA probe, which hybridizes the X chromosome. The arrow indicates ring form of the X chromosome.

Her case history and clinical information are given in Case 1. Another girl with primary amenorrhea, whose case history is given in Case 2, possessed a karyotype mos,45,XO/46,X+ringX. Of the 100 metaphases analyzed, 45 showed 45,XO (Figure 2(a)) and 55 showed 46,X+ringX karyotype (Figure 2(b)). X chromosome present in 45,XO karyotype was intact, whereas one of the X chromosomes present in 46,X+ringX chromosome complement showed the X chromosome in the form of a ring. The ring of the X chromosome was further confirmed by FISH using X-chromosome-specific painting probe (Figure 2(c)).

**Figure 3:**
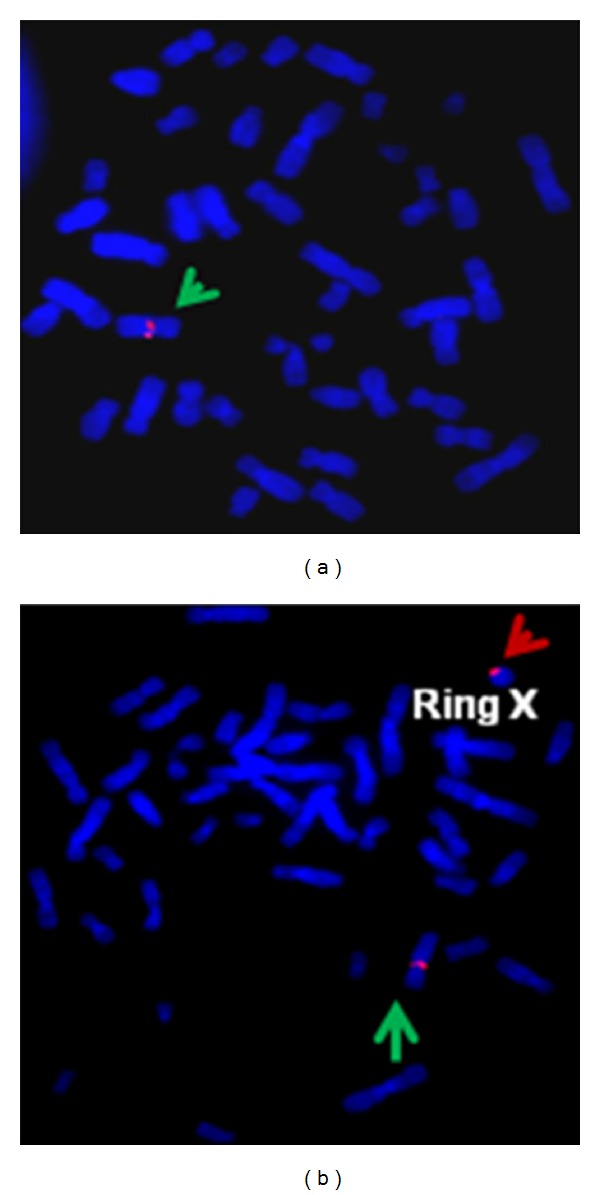
FISH of metaphase spread with the Vysis LSI AR DNA probe, which hybridizes the X chromosome q11-12. Green arrows indicate normal X chromosome, whereas red indicates ring form of the X chromosome. (a) 45,XO karyotype showing androgen receptor signal. (b) Androgen receptor signals in metaphase with 46,X+ringX karyotype.

The presence of Androgen Receptor locus (Xq11-12) of the X chromosome was confirmed by FISH using Locus-Specific Painting probe. The androgen receptor signals are seen in normal X and also in ring form of the X chromosome as shown in Figures 3(a) and 3(b).

**Figure 4:**
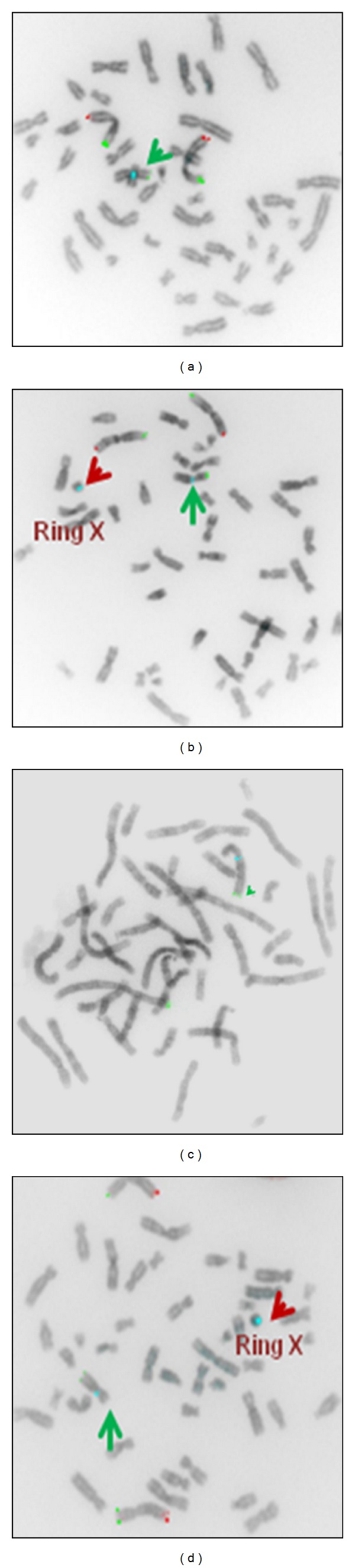
FISH of metaphase spread with the Vysis Telvysion probe, which hybridizes the Xp and Xq telomeres signals (a) and (c) shows Xp and Xq telomere signals on 45, XO metaphase spread, whereas (b) and (d) show Xp and Xq signals on normal chromosome X, and loss of Xp and Xq on 46,X+ringX metaphase spread.

The telomeres of the normal and ring X chromosome were studied using Mixtures 1 and 2 from ToTelVysionTM multicolor-color DNA Probe Mixtures from Vysis. *Mixture 1 *(Figures 4(a) and 4(b) TelVysion 1p SpectrumGreen, TelVysion 1q SpectrumOrange, TelVysion Xp/Yp SpectrumOrange and SpectrumGreen, and CEP X Spectrum Aqua showed SpectrumGreen signals for telomeres on Xp regions of normal X chromosomes and no signals on ring X chromosome. SpectrumGreen and SpectrumOrange signals are seen on chromosome 1p and 1q regions as well since the Mixture 1 contains the combination of probes for chromosomes 1p, 1q and Xp telomeres. *Mixture 2* (Figures 4(c) and 4(d)) TelVysion 2p SpectrumGreen, TelVysion 2q SpectrumOrange, TelVysion Xq/Yq SpectrumOrange and SpectrumGreen, and CEP X SpectrumAqua showed SpectrumGreen signals for telomeres on Xq regions of normal X chromosomes and no signals on ring X chromosome. SpectrumGreen and SpectrumOrange signals are seen on chromosome 2p and 2q regions as well since the Mixture 2 contains the combination of probes for chromosomes 2p, 2q and Xq telomeres. Both normal and ring X chromosome showed SpectrumAqua signals at their centromeric regions with Mixtures 1 and 2. Loss of telomeres was seen in the ring form of X chromosome with no signals using Mixtures 1 and 2 thus indicating that the sticky ends must have led for the formation of ring.

## 4. Discussion

 Cytogenetic analysis of women with ovarian defects revealed that the chromosome abnormalities are associated with 14% of the analyzed cases. Interestingly, among the majority of the abnormalities, approximately 70% of the defects involved the X chromosome in the form of 45,XO or variant. Mechanisms underlying the chromosome defects have been reported earlier [7, 12, 13]. Loss of X chromosome (Turner's syndrome) is a result of either anaphase lag or mitotic nondisjunction in the early embryonic stage [14–16]. The typical phenotypic changes observed were short stature, webbing of neck, shield chest, cubitus valgi, and absence of secondary sexual characters. Ultrasonographic examination of majority of cases revealed that the uterus is not visualized, and a few showed hypoplastic uterus. Other types of defects observed include testicular feminization (primary amenorrhea) with 46,XY karyotype in 12 individuals. The X-Y interchange hypothesis states that at meiosis in males, interchanges between pairing areas of the X and Y chromosomes could produce X chromosome that contains some Y material or a Y chromosome that contains some X material [17]. These XY females display gonadal dysgenesis. It has also been found that different portions of the chromosome could overlap thereby causing the variation in the severity of the sexual disorders [18]. X-chromosome mosaicism with 45,XO/46,XX karyotype may be the consequence of elimination of one of the X chromosomes during the subsequent divisions of zygote that is initially with a normal female chromosome complement [19]. Mos,46,XX/46,XY karyotype is a product of double fertilization by X-and Y-bearing sperms [20]. Although there are several reports on the occurrence of mosaicism [9, 21], this is the first report of two unique structural abnormalities, involving X chromosome in association with ovarian failure.

 An interesting karyotype associated with ovarian failure was 46,XX,del(Xq21-22) and q28. In addition to secondary amenorrhea, the proband was also found to have *b*lepharophimosis *p*tosis and *e*picanthus inversus *s*yndrome (BPES). Earlier studies suggested that the BPES was associated with the autosomal abnormality, involving 3q23 and 7p13 [22–24]. By linkage analysis, critical region for BPES has been mapped at 3q22-q23 [25]. However, this is the first report of BPES phenotype associated with X chromosome abnormality. The explanation could be either that BPES phenotype is a multifactorial, or the sequence presents between 3q22-q23 has homology with sequence at Xq21-q22 and/or Xq28. However, so far, six putative genes for ovarian function have been identified. Of these, DIAPH2 and FMR1 genes were localized on Xq22 and Xq28, respectively [26]. As the abnormality observed in this study involved both the regions (Xq22 and 28) suggesting that the ovarian defect in this case might be due to this deletion. There is evidence that the sex chromosomes (X and Y) have homologous sequence on autosomes [27, 28]. Molecular mechanisms of the X-chromosome deletions in familial cases are well documented [29, 30]. Deletion between Xq21-q22 in this case may be due to recombination between homologous chromosomes during the meiosis, most probably in mother. During the recombination, one of the X chromosomes might have gained the additional copy of Xq21-q22. When the egg carrying the X chromosome with the deletion of Xq21-q22 is fertilized with the normal sperm, it would have led to this abnormality.

 Another abnormality associated with primary amenorrhea was mos,45,XO/46,X+ringX that had both structural and numerical abnormality (Figures 2(a) and 2(b). Mosaicism of 45,XO and 46,X+ringX was found in 1 : 1 ratio. The structural abnormality of ring formation of one of the X chromosome was found only in metaphase possessing 46,Xr(X) chromosome complement (Figure 2(b)). This unique abnormality may be due to the occurrence of the deletion during gametogenesis in one of the parents followed by nondisjunction in the early stage of the embryonic development. Ring X chromosomes have been recognized in girls with Turner syndrome, often with mosaicism for a 45,XO cell line. In some instances, they are associated with mental retardation and a distinct phenotype of short stature, facial dysmorphism characterized by long palpebral fissures, a relatively broad nasal root and tip, anteverted nares, a wide mouth with a thin upper lip, soft tissue syndactyly, and mental handicap. Careful cytogenetic characterization of the ring X chromosomes has suggested that the smaller the size of the ring X, the more likely are the findings of mental handicap and dysmorphic features in the patient. This has been attributed to failure of dosage compensation, by X chromosome inactivation, for the genes on the ring chromosome [31–33].

A region of the proximal long arm of the X chromosome is required in cis for inactivation of the chromosome [34, 35] and contains the XIST gene, which is expressed exclusively from the inactive X chromosome [36] and is necessary for X inactivation in mice [37]. Smaller ring chromosomes may lack the XIST locus, rendering them functionally disomic for the genes present on the ring [38, 39]. The phenotype of individuals with small ring X chromosomes presumably results from the continued expression of genes in the pericentromeric region of the X chromosome due to failure of inactivation [8].

Patients with Turner syndrome present with a variety of phenotypes. However, in most X-chromosome abnormalities, a preferential inactivation of the abnormal X occurs, and this results in a mild phenotype [40]. Small ring X chromosomes lacking the XIST locus (Candidate Gene for X-inactivation Center) may not be subject to X inactivation and therefore present with a greatly affected phenotype [40]. Our patient presented with primary amenorrhea showed Turner phenotype, probably due to the equal presence of 45,XO cells along with the mosaic pattern. However, we did not investigate the presence of XIST in the cells that showed.46, Xr(X) karyotype.

Very few cases with X chromosome and autosome translocations have been reported [41]. Chromosome 3, 9 and 15 are the frequent partners of X-autosome translocations [42]. Here in our study, we report autosome defects at a very low frequency that include chromosomes 2, 3, 4, 7, 9, 15, 17, and 19 involved in translocations, inversions and deletions that are associated abnormal ovarian development and function.

To summarize, our study showed novel X-chromosome abnormalities associated with ovarian defects, and these observations would be helpful for genetic counseling. Besides, infertility clinics could use them to decide suitable strategies to help patients. The sequence analysis of the breakpoints of both autosomes and X chromosomes involved could help in understanding the molecular basis of ovarian defects and to map the genes involved.

##  Conflict of Interests 

The authors declare that they have no competing interests.

##  Authors' Contribution

L. Rao, V. Padmalatha, M. Kanakavalli, M. Swapna, M. Vidyadhari, G. Saranaya, K. Himaja, and L. Singh from Centre for Cellular and Molecular Biology, Hyderabad, India helped in chromosome analysis of the patients. M. Deenadayal, B. Sethi, P. Deb, N. Gupta, B. Chakraborthy, and P. Nallari from the other affiliations helped in clinical diagnosis and recruitment of patients for the study.

## Figures and Tables

**Table 1:** Cytogenetic findings in 53 cases with abnormal X chromosome.

No of cases	Karyotype	Diagnosis	Clinical features
12	45,XO	Primary amenorrhea	Short stature, small ovaries, growth failure, facial dysmorphic features
12	46,XY female	Primary amenorrhea	Short stature, gonadal dysgenesis, ovaries not visualized
3	46,X.i(X)(q10; q10)	Primary amenorrhea	Low set ears, low hairline at the back of neck
4	mos 45,XO/46,X iso(Xq)	Primary and secondary amenorrhea	Short stature
3	mos 45,XO/47,XXX	Primary amenorrhea, POF	Failed extensive treatment
2	mos 45,XO/46,XO+mar	Primary amenorrhea	Short stature
1	mos 45,XO/47,XX+mar	Secondary amenorrhea	Short stature
2	mos 45,XO/46,X,r(X)(p22.3q28)	Primary amenorrhea	Short stature
3	mos 46,XX/47,XXX	Secondary amenorrhea, POF	Failed extensive treatment
3	mos 45,XO/46,XY	Primary amenorrhea	Short stature
1	46,XX del(q21-22) and q28	Secondary amenorrhea	BPES^†^
2	46,Xdel(X)(q22>qter)	POF, secondary amenorrhea	Short stature
1	mos 45,XO/46,Xdel(Xp)	Primary amenorrhea	Short stature
1	mos 45,XO/46,Xdup(Xp)	POF	Failed extensive treatment
1	mos 45,XO/46,dup(Xq)	Primary amenorrhea	Short treatment
1	mos 45,XO/46,XX	Primary amenorrhea	Small ovaries
1	mos 45,XO/46,XX/47,XXX	Primary amenorrhea	Small ovaries

53			

^†^Blepharophimosis ptosis epicanthus inversus Syndrome.
